# Hyperpolarization-Activated Cation Channels Shape the Spiking Frequency Preference of Human Cortical Layer 5 Pyramidal Neurons

**DOI:** 10.1523/ENEURO.0215-23.2023

**Published:** 2023-08-28

**Authors:** Happy Inibhunu, Homeira Moradi Chameh, Frances Skinner, Scott Rich, Taufik A. Valiante

**Affiliations:** 1 Division of Clinical and Computational Neuroscience, Krembil Brain Institute, University Health Network, Toronto, Ontario M5T 1M8, Canada; 2 Institute of Biomedical Engineering, University of Toronto, Toronto, Ontario M5S 3E2, Canada; 3 Electrical and Computer Engineering, University of Toronto, Toronto, Ontario M5S 3G4, Canada; 4 Institute of Medical Science, University of Toronto, Toronto, Ontario M5S 1A8, Canada; 5 Division of Neurosurgery, Department of Surgery, University of Toronto, Toronto, Ontario M5T 1P5, Canada; 6 Departments of Medicine, Neurology and Physiology, University of Toronto, Toronto, Ontario M5S 3H2, Canada

**Keywords:** computational neuroscience, cortex, frequency-dependent gain, h-channel, human neuron modeling, layer 5 pyramidal cell

## Abstract

Discerning the contribution of specific ionic currents to complex neuronal dynamics is a difficult, but important, task. This challenge is exacerbated in the human setting, although the widely characterized uniqueness of the human brain compared with preclinical models necessitates the direct study of human neurons. Neuronal spiking frequency preference is of particular interest given its role in rhythm generation and signal transmission in cortical circuits. Here, we combine the frequency-dependent gain (FDG), a measure of spiking frequency preference, and novel *in silico* analyses to dissect the contributions of individual ionic currents to the suprathreshold features of human layer 5 (L5) neurons captured by the FDG. We confirm that a contemporary model of such a neuron, primarily constrained to capture subthreshold activity driven by the hyperpolarization-activated cyclic nucleotide gated (h-) current, replicates key features of the *in vitro* FDG both with and without h-current activity. With the model confirmed as a viable approximation of the biophysical features of interest, we applied new analysis techniques to quantify the activity of each modeled ionic current in the moments before spiking, revealing unique dynamics of the h-current. These findings motivated patch-clamp recordings in analogous rodent neurons to characterize their FDG, which confirmed that a biophysically detailed model of these neurons captures key interspecies differences in the FDG. These differences are correlated with distinct contributions of the h-current to neuronal activity. Together, this interdisciplinary and multispecies study provides new insights directly relating the dynamics of the h-current to suprathreshold spiking frequency preference in human L5 neurons.

## Significance Statement

Understanding the contributions of individual ionic currents to neuronal activity is vital, considering the established role of ion channel modifications in neuropsychiatric conditions. We combine *in vitro* characterization of the spiking frequency preference of human L5 cortical pyramidal neurons via the FDG with new analyses of a biophysically detailed computational model of such a neuron to delineate the connection between the dynamics of the h-current before spiking and key properties of the FDG. By further determining that both these FDG properties and h-current dynamics are distinct in analogous rodent neurons, we provide convincing evidence for the key role of the h-current in the suprathreshold frequency preference of human L5 cortical neurons.

## Introduction

Discerning how ion channels contribute to neuronal output is a daunting but essential task, as specific channel types play key roles in neuropsychiatric conditions (Martinez-Losa et al., 2018; Chang et al., 2019; Sun et al., 2019; Lupien-Meilleur et al., 2021). This is particularly relevant to the study of the human brain considering its differences from preclinical models (Molnár et al., 2008; Verhoog et al., 2013; Mohan et al., 2015; Eyal et al., 2016; Deitcher et al., 2017; Beaulieu-Laroche et al., 2018; Boldog et al., 2018; Kalmbach et al., 2018, 2021). Such differences motivate ongoing characterizations of human cell types (Bakken et al., 2021; Kalmbach et al., 2021; Moradi Chameh et al., 2021), a challenging task given limitations on human data (Rich et al., 2022) and the relative paucity of cell and circuit mapping tools used in rodent studies. Exacerbating this challenge is that ion channel activity is typically characterized using voltage clamp, preventing the simultaneous quantification of neuronal output. Although the role of an ion channel can be inferred by comparing activity with and without channel blockade (Kostyuk et al., 1981; Berger et al., 2001; Yue and Huguenard, 2001; Berger et al., 2003; Gu et al., 2005; Thomas et al., 2007; Ascoli et al., 2010; Kalmbach et al., 2018; Moradi Chameh et al., 2021), these interpretations depend on the mechanism of blockade and potential compensatory interactions.

These considerations are especially pertinent when studying the oscillatory dynamics of the brain (Buzsáki, 2006). Understanding the genesis of these rhythms involves accounting for their specific frequencies, recording and analysis methods, disease, and functional correlates (Buzsáki and Draguhn, 2004; Buzsáki et al., 2012; Maris et al., 2016; Mably and Colgin, 2018; Adamantidis et al., 2019; Hanslmayr et al., 2019). Results from preclinical models, although invaluable, must be applied cautiously to the human brain (Mohan et al., 2015; Eyal et al., 2016; Beaulieu-Laroche et al., 2018; Kalmbach et al., 2018; Boldog et al., 2018; Deitcher et al., 2017; Molnár et al., 2008; Verhoog et al., 2013); indeed, studies from human cortical slices have identified important nuances in their oscillatory dynamics (Florez et al., 2015; McGinn and Valiante, 2014).

Spiking frequency preference is an important factor underlying rhythm generation and signal transmission in cortical circuits (Higgs and Spain, 2009), being indicative of the potential role of a cell in pacemaking, phase locking, and amplifying frequency-modulated inputs. Therefore, understanding the influence of ion channels on spiking frequency preference represents a significant contribution to understanding functionally important aspects of neuronal dynamics. Previous experimental work (Moradi Chameh et al., 2021) identified the frequency preference of human cortical layer 5 (L5) neurons via the frequency-dependent gain (FDG; Higgs and Spain, 2009). These neurons exhibit a primary FDG peak between 2 and 6 Hz and a secondary peak at approximately double that frequency, both of which dissipate following application of the h-channel blocker ZD-7288 (Moradi Chameh et al., 2021), comporting with the hypothesis that the h-current contributes to low-frequency oscillatory activity (Beaulieu-Laroche et al., 2018; Kalmbach et al., 2018; Hu et al., 2002; Das and Narayanan, 2017). However, what specific features of h-current activity underlie its relationship with these oscillations remains mysterious.

Here, we dissect this complex neuronal dynamic in human cortical pyramidal neurons by applying the FDG to computational models. We first show that a biophysically detailed model of a human L5 neuron (Rich et al., 2021) reproduces key features of the experimental FDG, despite these features not being a constraint in model generation. Instead, this model was primarily constrained by subthreshold h-current driven activity (Rich et al., 2021), meaning its reproduction of complex spiking activity quantified by the FDG represents a strong connection between the dynamics of the h-current and spiking frequency preference. Further supporting this conclusion is the similar response of the computational and experimental FDG to h-current blockade. By combining the Currentscape visualization tool (Alonso and Marder, 2019) with spike-triggered average (STA) analysis (Schwartz et al., 2006), we reinforce this connection by identifying distinctive properties of h-current activity contributing to FDG features.

We then conducted a similar exploration with rodent neurons, motivated by known differences between human and rodent h-currents (Rich et al., 2021; Moradi Chameh et al., 2021). If the h-current is of special importance to spiking frequency preference, we would expect a distinct FDG profile in rodent neurons; indeed, new patch-clamp experiments in rodent L5 cortical pyramidal neurons identified an FDG profile distinct from that of analogous human neurons. We confirmed that a model rodent L5 pyramidal neuron (Hay et al., 2011) echoes these differences, justifying further application of our Currentscape/STA analysis. This revealed differences in the contribution of the h-current before spiking associated with the distinct FDG of the rodent model; considering the differing kinetics of the h-current in the human (Rich et al., 2021) and rodent (Hay et al., 2011; Kole et al., 2006) models, this indicates that the distinct dynamics of human h-channels are necessary for the frequency preference of human L5 neurons.

In summary, this study combines experimental results with novel *in silico* analyses to connect unique dynamics of the h-current to the spiking frequency preference of human L5 cortical pyramidal neurons. This interdisciplinary, multispecies approach provides convincing evidence for the special effect of the h-current on the spiking frequency preference of these neurons.

## Materials and Methods

### Computational models of human and rodent neurons

This work analyzes two computational models of cortical L5 pyramidal neurons, one of a human neuron (Rich et al., 2021) and another of a rodent neuron (Hay et al., 2011). Both models are accessible via ModelDB at https://senselab.med.yale.edu/ModelDB (accession no. 266984 for Rich et al., 2021; accession no. 139653 for Hay et al., 2011). Our implementation of the rodent neuron model uses the parameters found in the L5bPCbiophys3.hoc file. Both models are implemented in the NEURON simulation environment (Carnevale and Hines, 2006) and contain a biophysically detailed morphology, including a soma and various apical/basilar dendritic compartments.

Both models contain 10 ionic currents with differing maximum conductances, as well as different passive properties. The only differences in the kinetics of the ion channels are in the h-channel, the focus of the work of Rich et al. (2021). For ease of reference we refer to these currents by how they are denoted in the code of these models; Na_Ta_t is a fast, inactivating sodium current; Nap_Et2 is a persistent sodium current; K_Pst is a slow, inactivating potassium current; SKv3_1 is a fast, noninactivating potassium current; SK_E2 is a small-conductance calcium-activated potassium current; K_Tst is a fast, inactivating potassium current; Ca_LVA is a low-voltage-activated calcium current; Ca_HVA is a high-voltage-activated calcium current; Ih is a nonspecific hyperpolarization-activated cation current; and Im is a muscarinic current. We also track the passive current, denoted as pas.

All *in silico* experiments are performed in the somatic compartments of the corresponding models.

### *In vitro* recordings

**Figure 1.**
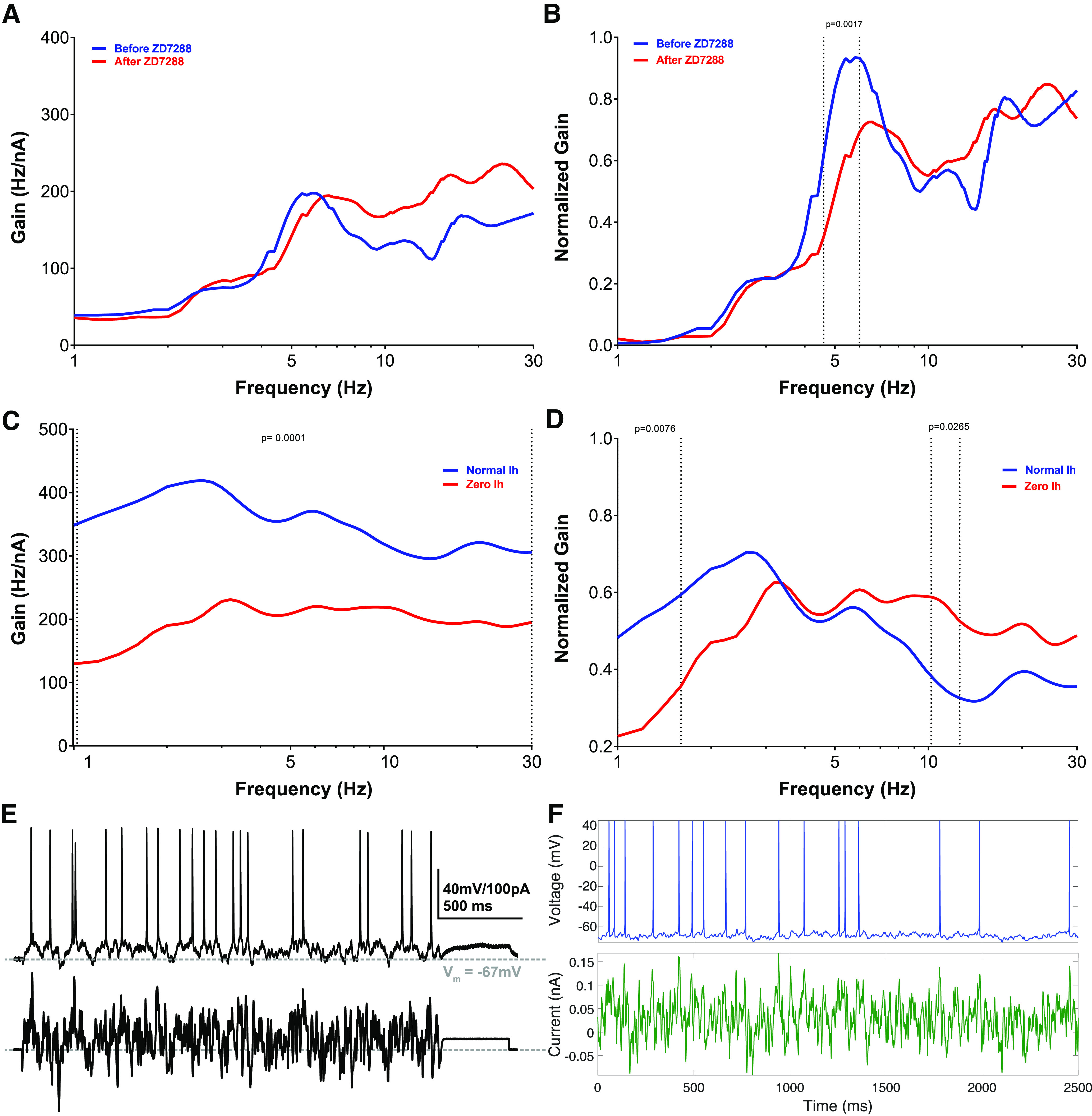
Model matches h-current-mediated FDG peaks observed experimentally in human layer 5 cortical pyramidal neurons. ***A***, ***B***, Experimentally calculated averaged non-normalized (***A***) and normalized FDG (***B***) of *n* = 3 L5 human pyramidal neurons with and without blockade of the h-channel with ZD-7288 (see above, Materials and Methods). Under control conditions, there is a clear low-frequency peak at ∼5 Hz. After treatment with ZD-7288, the peak FDG values occur at >10 Hz. Normalized curves exhibit significant differences (*p* = 0.0017) between 4.6 and 6 Hz, the location of this low-frequency peak. The mean SD of the non-normalized FDG curve is 26.849 before treatment with ZD-7288 and 63.094 after treatment with ZD-7288, and for the normalized FDG curve it is 0.172 before treatment with ZD-7288 and 0.145 after treatment with ZD-7288. ***C***, ***D***, Averaged non-normalized (***C***) and normalized FDG (***D***) derived from the model human L5 cortical pyramidal neuron under normal conditions and without h-current activity (see above, Materials and Methods). Normalized plots emphasize the qualitative correspondence between the model and experimental settings; the model exhibits a low-frequency peak (here at ∼3 Hz) under normal conditions, whereas this peak dissipates (yielding a flat FDG profile for >3 Hz) without h-current activity. Non-normalized plots are significantly different for 1–30 Hz with *p* = 0.0001; normalized plots are significantly different with *p* = 0.0076 for 1–1.6 Hz and with *p* = 0.0265 from 10–12.6 Hz. The mean SD of the non-normalized FDG curve is 76.170 under normal conditions and 71.779 with zero h-current activity, and for the normalized FDG curve it is 0.249 under normal conditions and 0.257 with zero h-current activity. Note that the normalized plots (***B***, ***D***) are not simply a rescaling of the absolute FDGs (***A***, ***C***), but are processed as defined (see above, Materials and Methods). All significance values are derived from the two-way ANOVA with Bonferroni’s multiple comparisons test. ***E***, ***F***, Example input current (bottom) and output voltage (top) traces in the experimental and model settings. ***E*** is replicated with permission from Moradi Chameh et al. (2021) while the default model setting is used in panel ***F***.

Data from human cortical L5 pyramidal neurons was obtained as described in Moradi Chameh et al. (2021). The data making up Figure 1*A, B*, is taken from the associated open source dataset (Howard et al., 2022) and reprocessed for presentation in a raw and normalized (see below) fashion. These data are from thin-tufted intratelencephalic (IT) pyramidal neurons from the middle temporal gyrus (MTG), as classified in Moradi Chameh et al. (2021). We also apply spike-triggered averaged analysis (STA) to the *n =* 3 human neurons in Moradi Chameh et al. (2021, used to create their Fig 5f) to qualitatively assess the correspondence between this measure in the *in vitro* and *in silico* settings, specifically the comparisons before and after the application of ZD-7288; this analysis had not been previously performed.

All experimental procedures involving mice were reviewed and approved by the Animal Care Committees of the University Health Network in accordance with the guidelines of the Canadian Council on Animal Care. Mixed male and female wild-type C57BL/6J, age postnatal 21 d, were used for experiments. Mice were kept on a 12 h light/dark cycle with food and water *ad libitum*.

Brain slice preparation was performed similarly for rodent tissue as outlined for human tissue in Moradi Chameh et al. (2021). Mice were deeply anesthetized by isoflurane 1.5–3.0%. After decapitation, the brain was submerged in (∼4°C) cutting solution that was continuously bubbled with 95% O_2_/5% CO_2_ containing the following (in mm): 248 sucrose, 2 KCl, 3 MgSO_4_.7H_2_O, 1 CaCl_2_.2H_2_O, 26 NaHCO_3_, 1.25 NaH_2_PO_4_.H_2_O, and 10 D-glucose. The osmolarity was adjusted to 300–305 mOsm. Mouse somatosensory cortical slices (350 μm) were prepared in the coronal plane using a vibratome (Leica 1200 V) in cutting solution. After slicing, slices were transferred to an incubation chamber filled with the following at 32°C (in mm): 126 NaCl, 2.5 KCl, 1.25 NaH_2_PO_4_.H_2_O, 26 NaHCO_3_, 12.6 glucose, 2 CaCl_2_.2H_2_O, and 1 MgSO_4_.7H_2_0, which was continuously bubbled with 95% O_2_/5% CO_2_. After 30 min, the slices were transferred to room temperature. Following this incubation, the slices were maintained in standard aCSF at 22–23°C for at least 1 h, until they were individually transferred to a submerged recording chamber (Moradi Chameh et al., 2021).

For electrophysiological recordings, cortical slices were placed in a recording chamber mounted on a fixed-stage upright microscope (Olympus BX51WI). Slices were continuously perfused with carbogenated (95% O_2_/5% CO_2_) aCSF containing the following (in mM): 123 NaCl, 4 KCl, 1.5 CaCl_2_.2H_2_O. 1.3 MgSO_4_.7H_2_O, 26 NaHCO_3_, 1.2 NaH_2_PO_4_.H_2_O, and 10 D-glucose, pH 7.40, at 32–34°C. Cortical neurons were visualized using an infrared charge coupled device camera (IR-1000, Dage-MTI) with a 40× water immersion objective. Patch pipettes (3–6 MΩ) were pulled from standard borosilicate glass pipettes (thin-wall borosilicate tubes with filaments, World Precision Instruments) using a vertical puller (PC-10, Narishige). For somatic recordings of electrophysiological properties, patch pipettes were filled with intracellular solution containing the following (in mm): 135 K-gluconate, 10 NaCl, 10 HEPES, 1 MgCl_2_, 2 Na2ATP, 0.3 GTP, pH adjusted with KOH to 7.4 (290–309 mOsm). Data were collected with excitatory (APV, 50 μm; CNQX, 25 μm, Sigma-Aldrich) and inhibitory (Bicuculline, 10 μm; CGP-35348, 10 μm, Sigma-Aldrich) synaptic activity blocked. ZD-7288 was applied through addition to the aCSF with a concentration of 10 μm.

Electrical signals were measured with an Axon MultiClamp 700A amplifier and the pClamp 10.6 data acquisition software (Molecular Devices). Subsequently, electrical signals were digitized at 20 kHz using an Axon 1440A digitizer (Molecular Devices). The access resistance was monitored throughout the recording (typically between 8 and 20 MΩ), and neurons were discarded if the access resistance was >25 MΩ.

For *in vitro* characterization of the frequency-dependent gain (see details below), a 2.5 s duration current stimulus of frozen white noise convolved with a 3 ms square function (Galán et al., 2008) was injected to each neuron 30 times for human neurons and 10 times for rodent neurons (with a 20 s intertrial interval). The statistics of this frozen white noise are an SD of 0.04 nA and *τ* = 3 ms, and it was created using the makeNoise.m MATLAB file included in our code repository. These statistics match those used in our previous work (Moradi Chameh et al., 2021), where they were chosen to ensure the noisy current was scaled to elicit spike rates of > 5 Hz, the typical firing rate for cortical pyramidal neurons. The firing rate under this experimental paradigm is additionally controlled by the choice of DC input, chosen specifically for each cell and experimental condition (i.e., before/after application of ZD-7288), to elicit ∼12–15 spikes in the 2500 ms experimental paradigm. The FDG of the neuron is the average of the FDGs calculated for each trial; the design of this stimulus is motivated in part by the previous literature (Padmanabhan and Urban, 2010) as well as its utility in our own previous work (Moradi Chameh et al., 2021). Data from *n* = 6 rodent neurons are shown (see Fig. 6), with these neurons classified as thin-tufted IT pyramidal neurons of the somatosensory cortex, given electrophysiological and morphologic similarities to our human neurons.

We emphasize here that an identical frozen white noise is used for each trial and for each neuron, whereas the DC shift is varied between neurons and dependent on application of ZD-7288 to compensate for varying intrinsic excitabilities. This ensures that the FDG is calculated for neurons with a similar mean firing frequency in the approximate theta range, with ∼12–15 spikes in the 2500 ms experimental protocol.

### *In silico* experimental protocol

*In silico* experiments were designed to mimic those performed *in vitro*. Thus, all *in silico* results are analyses of a current-clamp injection of white noise input (generated using the makeNoise.m MATLAB file included in our code repository with *σ* = 0.04 nA and *τ* = 3 ms) plus a tonic DC shift chosen to elicit ∼12–15 spikes in the 2500 ms experimental paradigm. In the human model, these experiments were performed in four scenarios, with the normal Ih maximum conductance as defined in Rich et al. (2021) (maximum somatic conductance of 5.14e-05 S/cm^2^), with that conductance doubled, with that conductance halved, and with that conductance zeroed (modeling channel blockade). Spikes were detected using a voltage threshold of greater than or equal to −55 mV for spike-triggered average analysis. Given the deterministic nature of the *in silico* model, our computational analyses involve injecting 30 different white noise inputs (with the same statistics) into the model neuron and averaging the resulting FDGs (see details below).

The DC shift for the human model was determined independently for each scenario given the effect of the h-current on the excitability of the model. These values were 0.003 nA for the scenario with doubled Ih maximum conductance, 0.031 nA for the default scenario, 0.040 nA when the maximum conductance was halved, and 0.073 nA when the maximum conductance was zero. In the rodent model, the DC shift was 0.38 nA both for the default model and the model with no h-current activity. The notable difference between the DC shift needed in the human and rodent models is reflective of the differing excitabilities of these models under this experimental protocol. Although we would not expect a direct correspondence between the DC shifts in the *in vitro* and *in silico* settings for multiple reasons, including the effects of cell-to-cell variability (Golowasch et al., 2002; Marder and Goaillard, 2006) and the differing model development techniques used in Rich et al. (2021) and Hay et al. (2011), the increased DC shift necessitated *in silico* with decreased *Ih* maximum conductance in the human model reflects a similar trend before and after application of ZD-7288 in human neurons *in silico*. The *in silico* DC shifts are also of similar orders of magnitude as those delivered *in vitro* in each setting.

### Frequency-dependent gain

This work presents an in-depth investigation of the FDG profiles of cortical L5 pyramidal neurons in the human and rodent setting both *in silico* and *in vitro*. The methodology follows that of Higgs and Spain (2009), adapted to the *in silico* and *in vitro* settings as described above. This measure is used to quantify the propensity of a neuron to fire in phase with an oscillatory input of small magnitude relative to the overall input to the cell or, in essence, to track particular frequencies in oscillatory input. The physiological implications of this measure include viable explanations for the oscillatory frequency preference of cortical regions as outlined in detail in our previous work (Moradi Chameh et al., 2021). We note that this measures a distinct element of the frequency preference of a neuron compared with other common measures, such as subthreshold resonance in response to a ZAP input (Hutcheon and Yarom, 2000; Rich et al., 2021; Moradi Chameh et al., 2021).

After detecting action potentials, a time varying firing rate *r*(*t*) is calculated to be the following:

$$r(t)=\{\begin{array}{ll} \frac{1}{\Delta t}, & \text{Where spikes detected}\\ 0, & \text{Otherwise} \end{array}.$$

The window Δ*t =* 0.00001 s in our experimental data, matching the sampling frequency. This same Δ*t* is used in our analysis of the human model neuron, whereas $\Delta t=0.0000\bar{3}$ s is used in our analysis of the Hay model, given computational limitations (see below).

Then, the stimulus–response correlation (*c_sr_*) and the stimulus autocorrelation (*c_ss_*) is calculated via the following:

$$c_{sr}(\tau )=\langle s(t)r(t+\tau )\rangle$$

$$c_{ss}(\tau )=\langle s(t)s(t+\tau )\rangle$$

for *τ* the time difference and *s*(*t*) representing the noisy stimulus.

The complex Fourier components *C_sr_*(*f*) and *C_ss_*(*f*) are then obtained, and the frequency-dependent gain is calculated as follows:

$$G(f)=\frac{\left|C_{sr}(f)\right|}{\left|C_{ss}(f)\right|}.$$

The FDG is calculated at discrete frequencies, with 0.2 Hz steps between 1 and 30 Hz. In this work we present the FDG between 1 and 30 Hz given our specific interest in the preference of a neuron for spiking that tracks low-frequency inputs, as well as to maximize the correspondence between presentations of our new *in silico* and previous *in vitro* data. Future work using the tools presented in this work might prove useful in discerning relationships between specific ionic currents and spiking frequency preference for inputs at higher frequencies.

In both the *in silico* and *in vitro* settings, we compensated for varying intrinsic neuronal excitabilities between neurons and in response to treatment with ZD-7288 by varying the DC current injected along with our white noise input. This follows the approach described in our previous experimental work (Moradi Chameh et al., 2021), motivated by the existing literature (Padmanabhan and Urban, 2010), which identified a consistent spiking frequency preference using the FDG in similarly classified human neurons. Considering these positive past results, we would expect our technique for controlling for differing excitability levels to yield similar results to other contemporary techniques that instead adjust statistics of the noisy input between neurons (Newkirk et al., 2022).

#### Averaging, normalization, and statistical tests

The averaged FDGs (see below, Results) are generated using subtly different processes in the *in silico* and *in vitro* settings. This choice is made considering that the *in silico* model is deterministic, whereas minor differences are expected on injection of an identical current input to the same neuron *in vitro* given the inherently noisy and stochastic nature of biological processes (Tripathy et al., 2013; Padmanabhan and Urban, 2010). The averaging for *in vitro* results follows the procedure outlined in our previous work (Moradi Chameh et al., 2021); we first average over the trial injections (of the same frozen white noise) to a given neuron under a particular experimental condition and then average again over the neurons (making this an average of averages). In the *in silico* setting, we average the FDGs generated from 30 trials injecting different white noise with the same underlying statistics.

Although this choice might potentially impair the exact correspondence between the *in silico* and *in vitro* settings, it does not affect the qualitative correspondence in the primary features of the FDG that are the focus of our analyses. We acknowledge that applying a single frozen white noise input to our neurons *in vitro* may inadvertently bias the results dependent on features of that frozen white noise, but this was necessary given the limitations on these recordings from human tissue. We indirectly address this concern by adjusting our protocol in the *in silico* setting, fully exploiting the benefits of a computational model to more thoroughly characterize its spiking frequency preference in response to multiple noisy inputs with similar statistics, which would be intractable in our *in vitro* recordings of human neurons.

The normalized FDG plots are correspondingly generated in subtly different fashions in the *in silico* and *in vitro* settings. We note that this normalization is not performed directly on the mean FDG curves (Fig. 1*A*,*C*) but on each individual FDG curve making up this average; this is why the normalized plots do not vary between zero and one. This approach was adopted to emphasize the key features of each FDG making up the average, namely the peaks representing a frequency preference, independent of differences in the magnitude of the overall gain that could vary among neurons/trials/settings. In the *in vitro* setting, we apply a standard normalization $\left(\frac{x-x_{min}}{x_{max}-x_{min}}\right)$ over the 1–30 Hz range on the averaged FDG for each neuron, and average across neurons to yield the presented normalized FDG. In the *in silico* setting, we apply this standard normalization to each trial (corresponding with a distinct white noise input) and average across trials to yield the presented normalized FDG.

The two-way ANOVA with Bonferroni’s multiple comparisons test is used to derive statistical significance between FDG curves. A standard cutoff of *p* < 0.05 is used to determine significance. Reported *p* values are averaged over the regimes with significant differences.

### Currentscape visualization

We apply the Currentscape visualization tool (Alonso and Marder, 2019) to display the dynamics of ionic currents as percentage of contributions over time using stacked-area plots. This tool provides an interface between the NEURON software, where the neuron model is situated and run, and Python software, which facilitates data visualization. An array of currents within the model is inputted into Currentscape and split into inward and outward currents. Inward currents are represented as negative values, whereas outward currents are represented as positive values. For each current, the percentage of its contribution to the overall inward/outward current at each time step is calculated.

During the simulation, the ionic currents, membrane voltage, and action potentials are extracted from the simulation in NEURON and imported into the Currentscape tool in Python. All code used in running these Currentscape visualizations are included in the code repository. Of note, whether the human (Rich et al., 2021) or rodent (Hay et al., 2011) model is implemented depends on whether the command h.load file(“init_final.hoc”) or h.xopen(“Hay_setup.hoc”) is invoked. We set h.cvode_active(0) to ensure a fixed time step for these simulations. The simulations performed in this study, motivated by the calculation of the FDG, involve calling the noiserun procedure with four inputs, the name of the file housing the noisy input, the value of *dt* (0.01 for human simulations, $0.0\bar{3}$ for rodent simulations), the simulation duration (2500 ms), and the DC shift (outlined above for differing Ih conductances).

Ultimately, Currentscape outputs a plot containing (1) voltage traces (mV); (2) total inward, outward, and total current traces (nA), and (3) percentage of contributions to the inward and outward current by each of the channels included in the model. Note that in this study the only compartment analyzed was the soma to best correspond with the *in vitro* experiments (Moradi Chameh et al., 2021).

Currentscape simulations on the human neuron model were performed using the Neuroscience Gateway (NSG), an online supercomputer (Sivagnanam et al., 2013). Identical code is used as outlined above and is included in our code repository for simulations on NSG, but it must be packaged in one ZIP file included in the data section of the supercomputer. Once uploaded, performing the simulations on NSG involves executing a Python file that includes Currentscape and NEURON, as indicated above, in a specific platform of NSG that has NEURON via a Python interface.

Because of compatibility issues, Currentscape simulations on the rodent neuron model could not be performed on NSG. Running these simulations on personal machines required a coarser temporal mesh because of computational limitations, that is, $dt=0.0\bar{3}$ ms as opposed to *dt =* 0.01 ms used with the human neuron. This difference does not confound the features of that curve (the locations of FDG peaks) nor the visualization and analysis of the corresponding Currentscape plots.

### Spike-triggered average analysis

Traditionally, spike-triggered average analysis (Schwartz et al., 2006) is used to quantify the average input current preceding spiking activity in a neuron, as was done in L5 human cortical pyramidal neurons by Moradi Chameh et al. (2021) using a 30 ms window. Here, we exploit the additional data afforded by Currentscape to perform analogous analysis on the percentage of contribution to the inward or outward current of individual channels preceding spiking in our neuron models. We analyze these dynamics both in the 30 ms window used by Moradi Chameh et al. (2021) and in longer 100 and 200 ms windows to ensure capture of the slow h-channel kinetics.

To perform these analyses, we first followed the *in silico* experimental protocol outlined above. We detected spikes and only considered those for which there was no other spike in the 30, 100, or 200 ms preceding it (as appropriate for the given time window). We averaged the dynamics of the percentage contributions of the various currents over the time window before each of these spikes for each of the 30 noisy input currents. In this way, we extend the often used experimental tool of spike-triggered averaging beyond the input current, using Currentscape, to describe the dynamics of individual ionic and passive currents in the moments before spiking. This allows us to directly discern whether any ionic current contributes uniquely to spike timing.

#### Quantification of STA dynamics

We performed an additional analysis to quantify the qualitatively different features of the STAs of Currentscape-derived percentage contributions to inward/outward currents. For a particular current *c*, the raw STA trace is denoted as $I_{STA}^{c}$. We process $I_{STA}^{c}$ by first normalizing it over the given time window, dividing each value by the maximum value for outward currents or minimal value for inward currents in said window. We then smooth the curve using the MATLAB built-in smooth function (MATLAB, 2019), yielding a processed trace, denoted $I_{*}^{c}$, that ranges between zero and one.

How $I_{*}^{c}$ changes approaching spiking reflects its relative contribution and subsequent control of the spiking process. In particular, we characterize the monotonicity of $I_{*}^{c}$. Mathematically, a monotonic function is one that either never increases or never decreases. Here, we quantify the monotonicity of $I_{*}^{c}$ by calculating the proportion of the time series that is increasing and decreasing; a time series that is either increasing or decreasing 100% of the time is purely monotonic, whereas one that decreases 50% of the time and increases the other 50% would be maximally nonmonotonic (i.e., it would be increasing and decreasing for equal proportions of the time window). To accomplish this, we calculate the difference quotient for each time step, yielding a new time series $dI_{*}^{c}$. The proportion of negative values in this time series yields the proportion of the $dI_{*}^{c}$ time series that is decreasing, and vice versa, which we use to draw conclusions about the monotonicity of the contribution of the current *c* to the inward/outward current before spiking.

### Data availability

All code used in this work is openly available at https://github.com/FKSkinnerLab/HumanL5FDG.

## Results

### Model replicates key features of experimentally derived FDG with and without h-current contribution

The FDG identifies the propensity of a neuron to spike in phase with a small oscillatory input relative to the net input of the neuron (Higgs and Spain, 2009). Experimentally, as shown in Figure 1*A*, *B*, human L5 cortical pyramidal neurons exhibit a primary FDG peak in a low-frequency range between 2 and 6 Hz, with a secondary peak at approximately double that frequency. Under h-current blockade with ZD-7288, these peaks dissipate and the gain mostly increases with increasing frequency. This is quantified by the significant difference (*p* = 0.0017) between 4.6 and 6 Hz in the normalized curves (achieved via processing the raw FDG data; see above, Materials and Methods), a range including the low-frequency peak. The coefficient of variation (SD divided by mean) of the normalized FDG in the range of statistically significant differences is <0.084 in the default setting and <0.437 after application of ZD-7288, with this increased variability further illustrating a loss of frequency preference. An example of the experimental traces used to derive the FDG is illustrated in Figure 1*E*.

Notably, the FDG profile of the human L5 model neuron presented by Rich et al. (2021), illustrated in Figure 1*C, D*, replicates key features of the experimental FDG; there is a low-frequency peak (here at ∼3 Hz) and a secondary peak at double this frequency. These peaks also dissipate when the h-current is blocked in the model neuron, including a flat FDG profile for >3 Hz. The raw FDG traces with and without h-current activity (Fig. 1*C*) are significantly different with *p* = 0.0001 for the entire frequency range. Normalized plots (achieved via processing the raw FDG data (see above, Materials and Methods) emphasize the presence (or lack thereof) of peaks that indicate a frequency preference; these are significantly different for 1–1.6 Hz with *p* = 0.0076 and for 10–12.6 Hz with *p* = 0.0265. The difference at >10 Hz is particularly notable as it shows that the decrease in gain at high frequencies in the default model, which serves to accentuate the peaks indicating frequency preference, is significantly diminished without h-current activity. The coefficient of variation of the normalized FDG in the range of statistically significant differences is <0.707 under normal conditions and <0.935 with zero h-current activity; differences in these variability measures between the experimental and computational settings are likely attributable to the different averaging and normalization techniques implemented to best use the data in these distinct settings (see above, Materials and Methods). An example of the model output under the paradigm used to calculate the FDG is illustrated in Figure 1*F*.

Considering the model neuron was constrained primarily by subthreshold voltage traces and not by spiking activity in response to a noisy input current (Rich et al., 2021), the correspondence between the primary qualitative features of the experimental and model FDG profiles is a nontrivial and important result. Given the focus of the modeling process in Rich et al. (2021) on capturing h-current activity, it stands to reason that this FDG correspondence is in part driven by the model’s accurate encapsulation of h-current dynamics. This conclusion is supported by the additional correspondence between the FDG profiles of the experimental neuron treated with ZD-7288 and the model neuron with no h-current activity. Although the similarities between the *in silico* and *in vitro* FDGs are not exact, this is to be expected given the model generation process described above, as well as the different processes for generating averaged FDGs (an average of averages over neurons and trials, with a single frozen white noise, in the *in vitro* setting vs averaging over different white noises, albeit with identical generating statistics, in the *in silico* setting; see above, Materials and Methods) necessitated by the deterministic nature of the model neuron. One discrepancy of note is the increased gain at high frequencies seen experimentally following the application of ZD-7288; although the decrease in gain at these frequencies is significantly diminished in the model without h-current activity, an overall increase in gain is not observed. This is likely a consequence of secondary effects of treatment with ZD-7288 (Poolos, 2004; Sánchez-Alonso et al., 2008; Chevaleyre and Castillo, 2002), including an increased input resistance, that are not encapsulated by simply removing h-current activity in the model neuron. Nonetheless, the correspondence between the primary FDG features in the control setting (low-frequency peak and secondary peak at approximately double the frequency) and with h-current blockade (suppressed low-frequency activity and lack of a notable decrease in gain at >10 Hz) justifies the use of this *in silico* model as a tool to better understand the contribution of the h-current to the FDG of the neuron.

### Currentscape visualization of noisy current injections highlights current contributions

**Figure 2.**
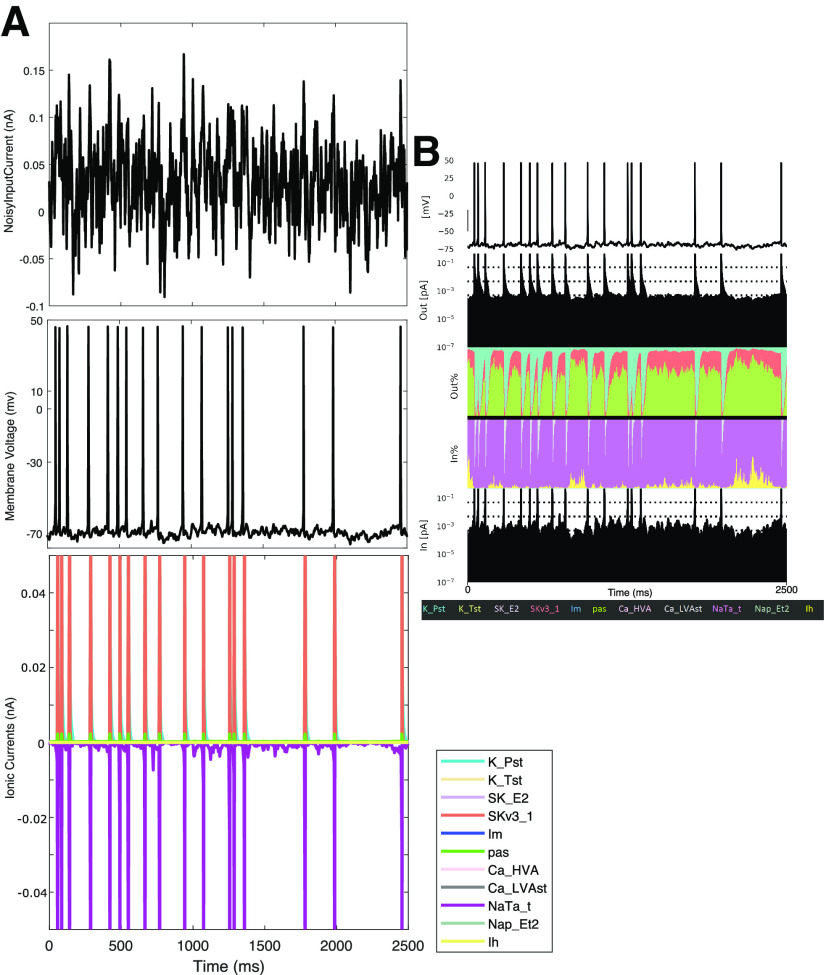
Currentscape visualization tool facilitates the identification of relative current contributions in spiking simulations. ***A***, Raw data obtained via NEURON of a simulation of a noisy input current injected into the L5 human pyramidal neuron model. Top to bottom: Noisy input current, voltage trace, and ionic current contributions. Of note is that the varying magnitudes of the ionic currents obscure one’s ability to jointly visualize their contribution to the dynamics of the neuron. ***B***, Using the Currentscape tool, the ionic current contributions are recontextualized as the outward and inward percentages of contribution plots, allowing for a more intuitive visualization on a single scale despite the spiking activity of the neuron. Note that Im is not discernible in this visualization, denoting its minimal contribution to the dynamics of the model in this scenario.

We sought to fully exploit the *in silico* setting to perform measurements that could not be done *in vitro*, simultaneously quantifying the activity of each ionic current alongside the voltage response to a noisy input current. Currentscape (Alonso and Marder, 2019) is invaluable for the visualization of these current contributions within spiking simulations. As illustrated in Figure 2*A*, current activity differs by orders of magnitude between neuronal spiking and subthreshold activity, obscuring the evolution of these contributions over the course of a simulation. Currentscape addresses this problem, as illustrated in Figure 2*B*, by instead calculating the percentage of the contribution of a given current to the net inward/outward current at a particular time step. This provides a more intuitive visualization of the evolution of ion channel contributions on a single scale despite the spiking dynamics of the neuron.

**Figure 3.**
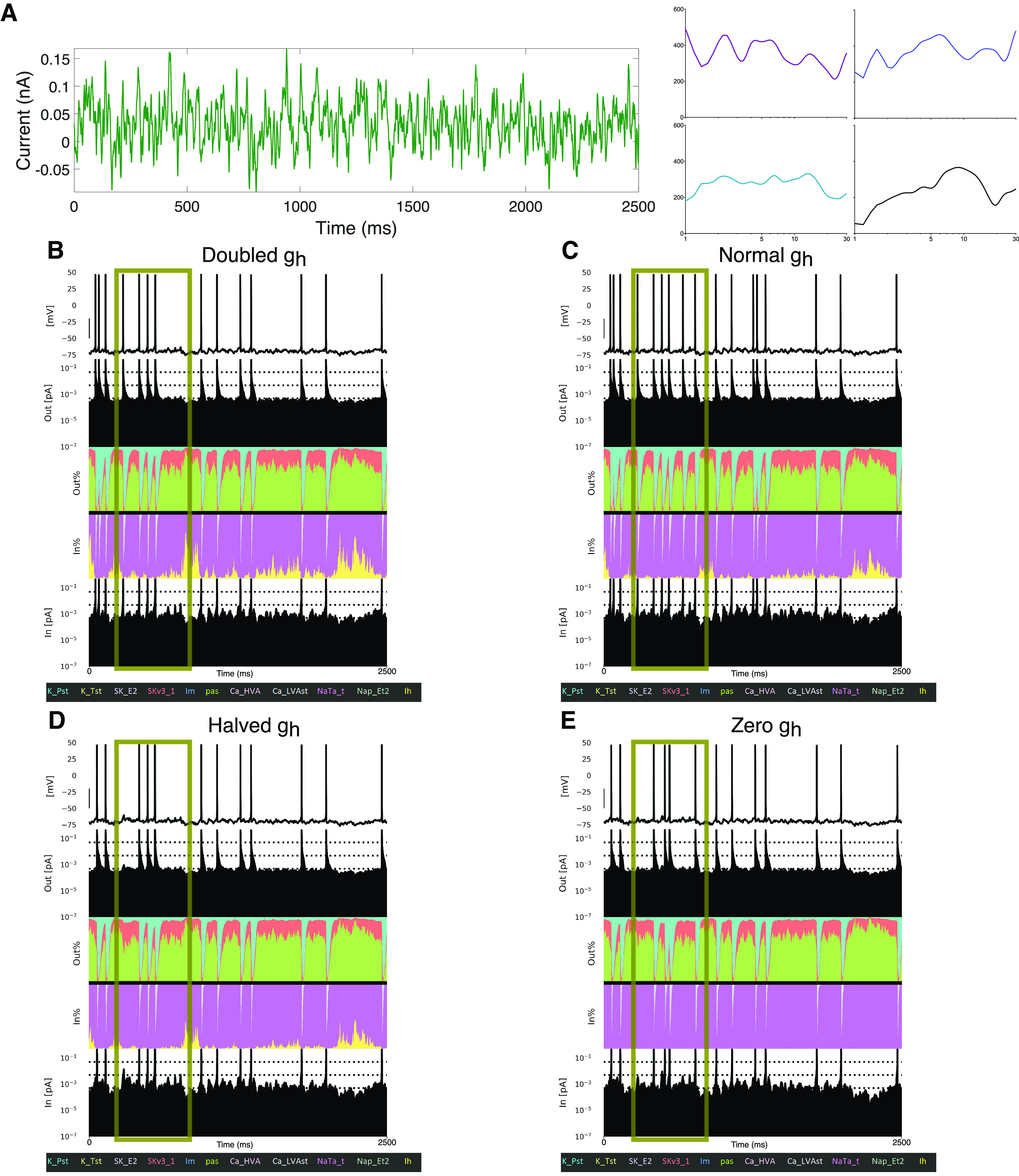
Currentscape visualization highlights differences in spiking modulated by h-current activity. ***A***, Left, The example noisy input current injected into the L5 human pyramidal neuron model with differing Ih conductance levels. Right, Example individual non-normalized FDGs [plot is Gain (nA) vs log-scaled frequency (Hz)] generated from this specific noisy input in each of the following scenarios: with the Ih maximum conductance doubled (top left, purple), normal (top right, blue), halved (bottom left, teal), or zero (bottom right, black). ***B***–***E***, Currentscape visualizations corresponding with each of the scenarios outlined above. The h-channel contribution is indicated in the inward current contribution of the visualization in yellow (fourth row, “In %”), alongside the contributions of all the ionic currents and passive currents in the model. Spiking activity differs in each scenario (see regime highlighted by the gold box) driven by the varying Ih conductance and the differing contributions of Ih to the activity of the neuron highlighted by this Currentscape visualization.

We used this tool to decipher and highlight differences in neuronal dynamics as the contribution of the h-current is changed (by altering its conductance value) in Figure 3. An example of the noisy input current used in these simulations is illustrated in Figure 3*A* (note the average value of this input, its DC Shift, is altered in each setting reflecting changes in the Ih maximum conductance; see above, Materials and Methods), and Currentscape plots are illustrated when the Ih maximum conductance is doubled (Fig. 3*B*), at its default value (Fig. 3*C*), halved (Fig. 3*D*), and zeroed (Fig. 3*E*). Currentscape visualization allows for dynamics of the h-current (yellow inward current) to be discerned that would otherwise be obscured by examining raw current magnitudes as in Figure 2*A*.

We highlight a regime in Figure 3*B*–*E*, with a gold box in which spiking differs in each scenario. Using Currentscape visualization, we can identify increased h-current activity at the end of this period when the Ih maximum conductance is doubled and when it is halved (Fig. 3*B*,*D*) compared with both the default state and when there is no h-current activity (Fig. 3*C*,*E*); in the former scenarios there is no spiking during this period, whereas in the latter scenarios there is. This nonlinear relationship among h-current activity, h-current conductance, and spiking highlights the complexity of these interactions in this biophysically detailed neuron model and motivates further quantifications in search of clearer relationships.

There are multiple ways one might quantify the differences in these spike trains as a result of the varying contributions of the h-current. One is the FDG itself; we include FDG plots of these specific example trials in Figure 3 to illustrate how FDG properties differ for differing spike trains. Differences are apparent from the FDGs of these individual trials, and the differences between FDGs in these scenarios averaged over multiple trails are statistically examined below. Another commonly used quantification of spike train features is the coefficient of variation (CV) of the interspike intervals (ISIs); although this is commonly measured in response to tonic current steps experimentally (Angelo and Margrie, 2011), here we apply this measurement to the response of our model to noisy input. We calculated the CV of the ISIs in each of the four scenarios for all 30 trials, and found the CV of the ISIs to be 0.78 for double Ih maximum conductance, 0.81 for normal Ih maximum conductance, 0.92 for halved Ih maximum conductance, and 1.05 for zero Ih maximum conductance. These CVs are significantly different between the doubled and halved Ih maximum conductance scenario, the doubled and zeroed Ih maximum conductance scenario, and the normal and zeroed Ih maximum conductance scenario (*p* < 0.05 in the first case, *p* < 0.001 in the last two cases; two sample coefficient of variation test). This pattern follows from the role of the h-current in dictating a spiking frequency preference; a neuron with a pronounced FDG peak will better track one of the many sinusoidal frequencies making up the white noise input, in turn firing more regularly and with less variability (smaller CV). This is borne out by the experimental data as well; analysis of the data yielding Figure 1*A, B*, shows that the CV of the ISIs is significantly increased following blockade of the h-current with ZD-7288 (0.413 vs 0.333; *p* = 0.00004, two-sample coefficient of variation test). The FDG and CV of the ISIs can thus be considered complimentary measures of a spiking frequency preference, representing further quantitative support for the notable differences in the spiking activity of the model neuron in response to varying Ih maximum conductance.

These relationships imply that the h-current affects more than the basic excitability features of the neuron (Dyhrfjeld-Johnsen et al., 2009; Gasselin et al., 2015; Hogan and Poroli, 2008); distinct spike trains, associated with distinct time-courses of h-current activity, are observed for varying Ih maximum conductances despite compensation for changes in excitability via the DC input. Indeed, the contribution of the h-current to neuronal spiking appears more complex, causing neurons with differing Ih maximum conductances to respond differently to the same noisy input, often in unintuitive ways (i.e., the lack of spiking toward the end of the highlighted region seen when the Ih maximum conductance is doubled and halved, compared with multiple spikes in the default setting).

Importantly, these conclusions would be difficult to directly establish using only contemporary *in vitro* experimental techniques and likely obscured without Currentscape visualization in the *in silico* setting. They remain, however, entirely qualitative. In the following, we further exploit the percentage contribution measure generated by Currentscape to quantitatively explore the relationship between h-current activity and spiking activity.

### STA analysis quantifies unique dynamics of the h-current before spiking

To quantify the contributions of each ionic current to spiking activity, we adapted STA analysis (Schwartz et al., 2006) by applying this technique to the percentage contribution of individual ionic currents to the net inward and outward current derived using Currentscape. In this process, we also confirmed that the traditional STA, as applied to the noisy current input as in Moradi Chameh et al. (2021), is qualitatively similar in our model and in the human neurons studied in Figure 1 *A, B*, (data not shown). This represents an additional validation that the model reasonably captures important features of the spiking dynamics of human L5 cortical pyramidal neurons.

**Figure 4.**
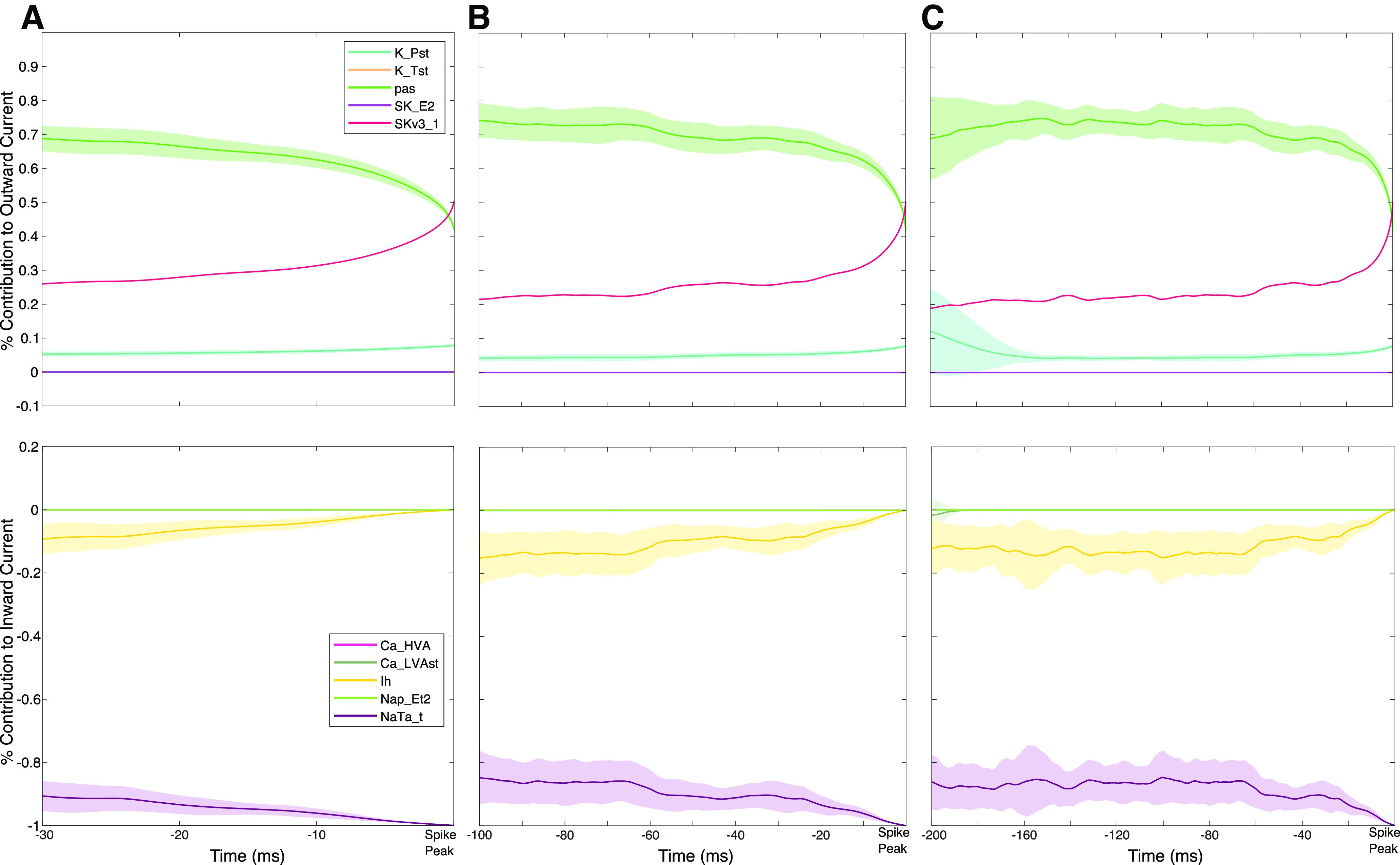
Currentscape data processing merged with spike-triggered average analysis of human cortical layer 5 neuron dynamics. ***A–C***, Representations of the STA for the percentage contribution to outward (top) and inward (bottom) currents for 30 ms (***A***), 100 ms (***B***), and 200 ms (***C***) before a spike. A unique dynamic is observed in the h-current activity in ***C***; over these 200 ms, the contribution of the h-channel is distinctively nonmonotonic. The shaded portion of each plot represents ± 1 SD over the 30 repetitions with distinct noisy inputs. Im is not included in these plots given its minimal contribution to model dynamics (Fig. 2).

**Figure 5.**
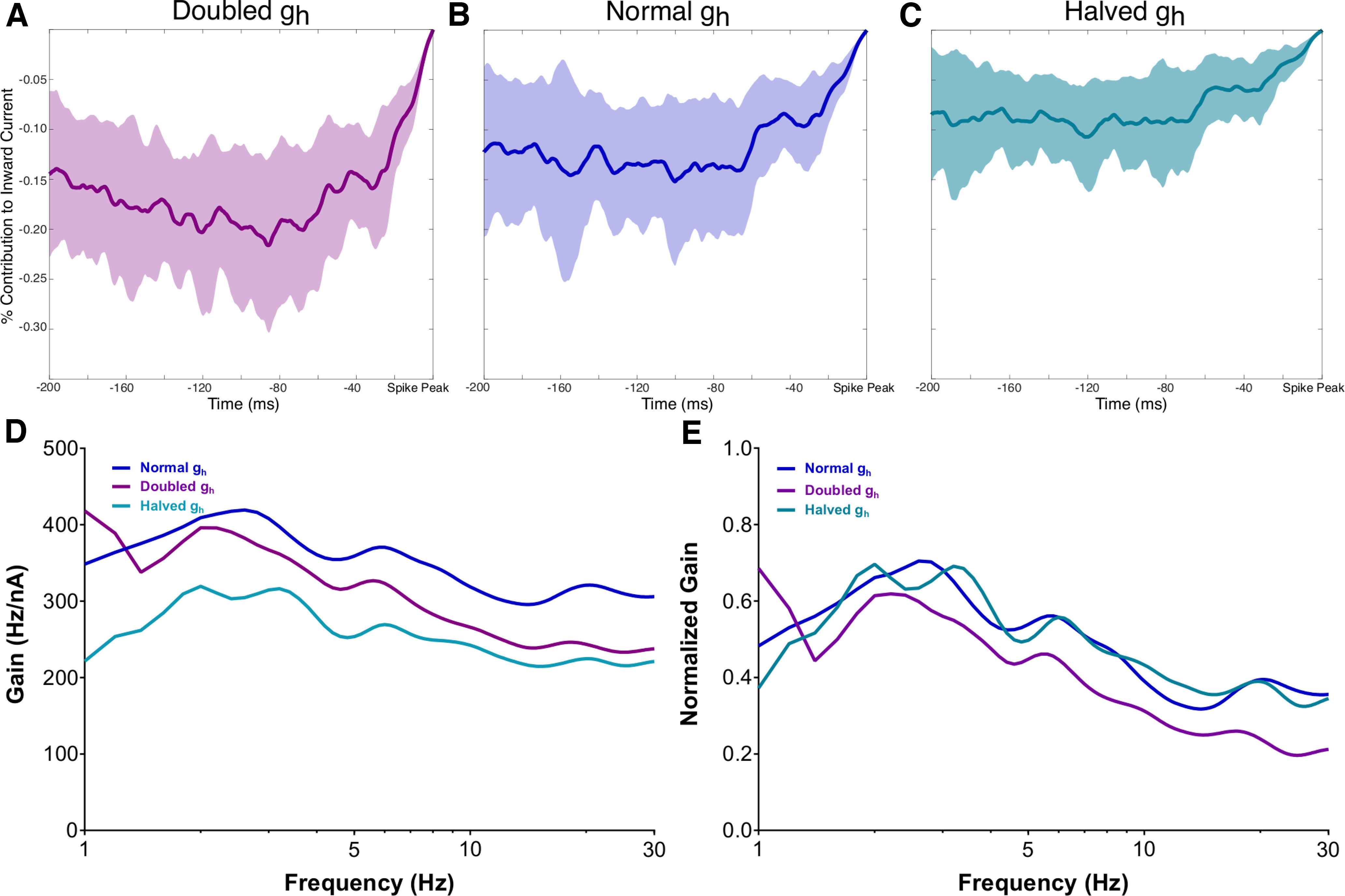
The nonmonotonic nature of h-current activity before spiking is exaggerated with an increased h-channel conductance and diminished with a decreased h-channel conductance. ***A–C***, Visualizations of the h-channel contributions preceding spiking with the Ih maximum conductance doubled in ***A***, normal in ***B***, and halved in ***C***, with the shaded regions representing ± 1 SD. ***D***, ***E***, Non-normalized (***D***) and normalized (***E***) average FDGs in each of the above scenarios (see above, Materials and Methods). The normalized plots highlight the flatter decay of the FDG at high frequencies when the Ih maximum conductance is halved and the more precipitous drop when the Ih maximum conductance is doubled. The former diminishes the influence of any low-frequency peaks, whereas the latter accentuates it. The mean SD of the non-normalized FDG curve is 76.170 under normal conditions, 74.763 when the Ih maximum conductance is doubled, and 72.422 when it is halved; for the normalized FDG curve the mean SD is 0.249 under normal conditions, 0.203 when the Ih maximum conductance is doubled, and 0.235 when it is halved.

We applied STA analysis to the percentage contribution of each individual current to the net inward/outward current at each time step, as quantified using Currentscape. We visualized this measure for 30 ms (Fig. 4*A*), 100 ms (Fig. 4*B*), and 200 ms (Fig. 4*C*) windows before spiking, with the larger time windows motivated by the slow kinetics of the human h-current (Rich et al., 2021). Differences in the dynamics of the h-current relative to other currents became apparent in the 200 ms before spiking, during which we see that the contribution of the h-current to the inward current is nonmonotonic; on average, the percentage contribution increases in the 200 and 100 ms before spiking but then decreases toward zero until the spike peak. (This monotonicity is further emphasized by the zoomed in plot in Fig. 5*B*.) In contrast, each of the outward currents behaves approximately monotonically for a larger proportion of the 200 ms time frame and exhibits more variability in the regime in which it does not; the pas current largely decreases, the K_Pst and SKv3_1 currents largely increase (the SKv3_1 current with notably minimal variability), and the K_Tst and SK_E2 currents both have a minimal, near zero percentage contribution. Meanwhile, among the inward currents, Ca_HVA, Ca_LVAst, and Nap_Et2 all have near-zero percentage contribution in the 200 ms time frame. This leaves the percentage contribution of the NaTa_t current inversely proportional to the percentage contribution of the h-current, as these currents together account for ∼100% of the inward current. Considering the h-current is maximally active at subthreshold voltages and acts on a slower time scale, whereas the NaTa_t current contributes primarily to action potential generation at a very fast time scale (Rich et al., 2021), we can reasonably assume that most changes in the inward current before spiking are driven primarily by the h-current.

Following the process outlined above in the Materials and Methods, we quantified the nonmonotonicity of the contribution of the h-current to the inward current visualized in the 200 ms STA. Given the relationship between the percentage contribution of the Ih and NaTa_t currents described above, we focused on comparing the 200 ms STA of the h-current to the 200 ms STA of the outward ionic currents; indeed, the primary ionic currents contributing to the outward current, SKv3_1 and K_Pst, both have quantitative distinctions from the dynamics of the h-current. The former current is almost entirely monotonic: $I_{*}^{SKv3\_1}$ is increasing for 94.72% of the 200 ms before spiking, in sharp contrast with the h-current, for which $I_{*}^{Ih}$ is decreasing for just 62.50% of the 200 ms before spiking. Meanwhile, there is a noticeable increase in the variability of the contribution of K_Pst between 200 and 150 ms before spiking (see the fainter ± SD shading of the cyan curve), a clear distinction in comparison to the other ionic currents. The remaining ionic currents either contribute minimally in the 200 ms before spiking (Ca_HVA, Ca_LVAst, Nap_Et2, K_Tst, SK_E2; also Im, which is omitted from the plots as mentioned in Fig. 3) or have a percentage contribution approximately inversely proportional to that of the h-current (NaTa_t). Thus, the qualitatively observed uniqueness of the 200 ms STA of the h-current relative to other ionic currents is supported by quantitative analysis.

Next, we applied our STA analysis to three of the scenarios studied in Figure 3; double, normal, and halved Ih maximum conductance. As can be seen in Figure 5*A–C*, the curvature of the STA of the percentage contribution of the h-current to the inward current is exaggerated when its conductance is doubled, and diminished when it is halved. We quantified this by examining $I_{*}^{Ih}$ in each scenario; we found that $I_{*}^{Ih}$ was decreasing for 70.37% of the STA time series when the Ih maximum conductance was halved, 62.50% of the STA time series for the default value of this conductance, and just 55.89% of the STA time-series when this conductance was doubled. This pattern confirms quantitatively what we observed qualitatively, that the monotonicity of these curves is affected by the amount of h-current activity, as dictated by the Ih channel maximum conductance. These changes, elicited solely by alterations to maximum conductance of the h-current, are also confirmatory evidence that the features of the percentage contribution of the NaTa_t current are most likely attributable to its relationship with the percentage contribution of the Ih current as discussed above.

These differences correspond with alterations to the corresponding FDGs, displayed non-normalized in Figure 5*D* and normalized in Figure 5*E*. Notably, when the Ih maximum conductance is halved, the low-frequency peak appears split, which undermines a key feature of the *in vitro* and default *in silico* FDG—the presence of a single low-frequency peak and single secondary peak at approximately double the frequency. This can also be seen in the individual FDGs in Figure 3*A*. Additionally, the decay of the gain at high frequencies is notably more gradual, potentially mitigating the functional frequency preference quantified by the FDG. Meanwhile, when the Ih maximum conductance is doubled, the decay of the gain at high frequencies is more precipitous, strengthening the frequency preference of the neuron at the low-frequency peaks. Details on the variability of these averaged FDG curves can be found in the Figure 5 legend.

The qualitative differences described above are confirmed via quantitative statistical testing. The normalized FDG when the Ih maximum conductance is doubled is significantly different from the default curve, for *p* < 0.05, for 19.2–30 Hz, whereas the non-normalized FDGs are significantly different for 6.4–30 Hz. Meanwhile, the non-normalized FDG when the Ih maximum conductance is halved is significantly different from the default curve, for *p* < 0.05, for the entire 1–30 Hz range.

We note that the idiosyncratic maximum gain at 1 Hz when the Ih maximum conductance is doubled is a likely side effect of the increased excitability of the model in this scenario (the DC shift necessary to elicit appropriate spiking is smaller by an order of magnitude when the Ih maximum conductance is doubled as compared with the default model; see above, Materials and Methods). Although this might be compensated for by changing other elements of the model neuron, we consciously chose to only manipulate the h-current to minimize any potential confounds. This nuance emphasizes the need to appropriately contextualize the conclusions drawn when altering model neurons such as these (Rich et al., 2021, hybrid model neurons).

This analysis reveals a correlation between the time course of the contribution of the h-current to the inward current before spiking and features of the FDG. By limiting our alterations to the model neurons only to the Ih maximum conductance, we can confidently conclude that the activity of the h-channel is responsible for these changes. In turn, we can directly infer a relationship between the expression of the h-channel (as quantified by the Ih maximum conductance), features of the activity of the h-current before spiking, and the presence (or lack thereof) of key features of the FDG of human L5 cortical pyramidal neurons.

### Comparison to rodent models identifies effect of h-current kinetics on FDG profile

The above analysis uncovers a relationship between the distinctive contribution of the h-current to neuronal dynamics before spiking and the features hallmarking the FDG of human L5 cortical pyramidal neurons. We obtained this relationship by directly comparing the output of the human neuron model and analogous experimental outputs expressing complex dynamics, combined with novel analyses of the dynamics of the model. Given this and the known differences between rodent and human cortical neurons, we wondered whether this relationship would be preserved for rodent cortical pyramidal neurons. Although a biophysically detailed model of a rodent L5 pyramidal neuron is available (Hay et al., 2011), an experimental quantification of the FDG of these neurons was not. We thus performed new *in vitro* explorations.

**Figure 6.**
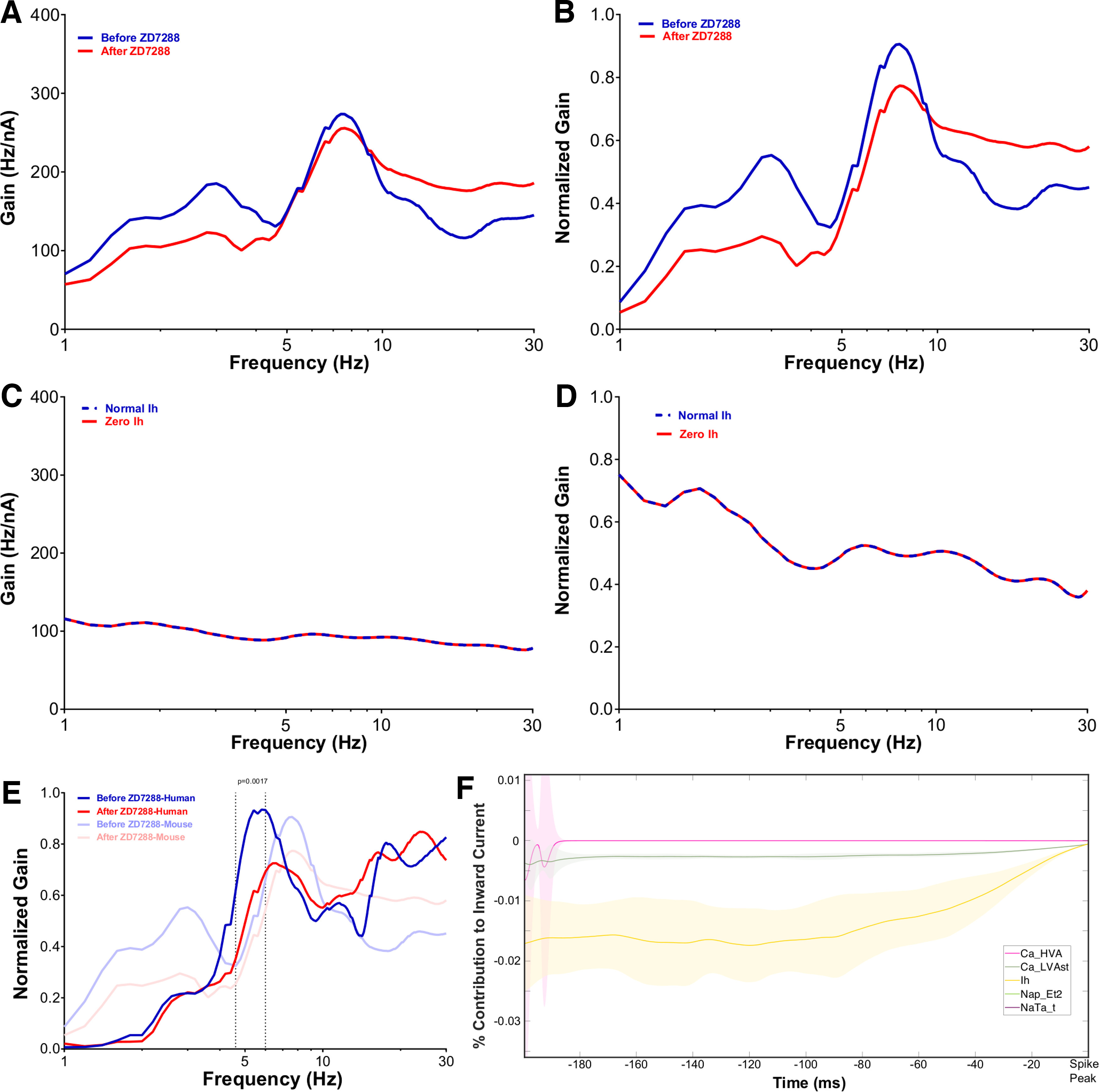
FDG properties are distinct in rodent L5 cortical pyramidal neurons, both *in vitro* and *in silico*, corresponding with a distinct h-channel contribution. ***A***, ***B***, Non-normalized (***A***) and normalized (***B***) FDGs averaged from *n =* 6 rodent L5 cortical pyramidal neurons firing approximately in the theta frequency range. The mean SD of the non-normalized FDG curve is 55.769 before treatment with ZD-7288 and 66.734 after treatment with ZD-7288, and for the normalized FDG curve it is 0.224 before treatment with ZD-7288 and 0.265 after treatment with ZD-7288. ***C***, ***D***, Non-normalized (***C***) and normalized (***D***) FDG analysis of the rodent L5 model of Hay et al. (2011) captures key qualitative properties displayed experimentally, including the lack of a peak in the 2–6 Hz range and minimal change when the h-current is blocked (here the FDG curves are identical), both stark contrasts from the human setting *in vitro* and *in silico*. The mean SD of the non-normalized FDG curve is 28.343 under normal conditions and 28.343 with zero h-current activity, and for the normalized FDG curve it is 0.261 under normal conditions and 0.261 with zero h-current activity. ***E***, Comparison between the normalized FDGs of human and rodent L5 cortical pyramidal neurons highlights their differences; these are statistically significant before treatment with ZD-7288 with *p* = 0.0017 between 5 and 5.4 Hz (2-way ANOVA, Bonferroni’s multiple comparisons test). ***F***, STA analysis of the inward currents for the default model of Hay et al. (2011) (magnified to emphasize the contribution of the h-current). In comparison to the human model, the contribution is much smaller in magnitude and notably more monotonic.

These experiments reveal clear differences between the FDGs of rodent and human L5 cortical pyramidal neurons; notably, the primary peak in rodent L5 pyramidal neurons occurs at ∼8 Hz rather than between 2 and 6 Hz in human neurons. Additionally, there are no significant differences between the curves before and after the application of ZD-7288 in rodent neurons (Fig. 6*A*,*B*). These interspecies differences are emphasized in Figure 6*E*, including significant differences between the settings before the application of ZD-7288 around the low-frequency peak of the human neurons. Along with the disruption of this low-frequency peak, there is also increased variability in the rodent setting in the 4.6–6 Hz frequency range indicative of a lack of frequency preference; the coefficient of variation is <0.762 before application of ZD-7288 and <0.791 after application of ZD-7288 over this range in the normalized curves. These similar variabilities also underscore the mitigated effect of ZD-7288 on the FDG in the rodent setting.

The FDG of the rodent model of Hay et al. (2011) preserves some key differences between the human and rodent settings seen via the experimentally quantified FDGs (Fig. 6*C,D*). Most notably, the FDG peak in the 2–6 Hz range, which is the most prominent feature in the human setting both *in vitro* and *in silico*, is entirely absent; in fact, the computationally derived FDG from the model of Hay et al. (2011) yields a qualitatively flat FDG with only a moderate decrease in gain with increasing frequency, indicating minimal suprathreshold frequency preference in this neuron model. The lack of any difference with and without h-current activity also mirrors the diminished effect of ZD-7288 on experimentally derived rodent FDGs. Details regarding the variabilities in the normalized and non-normalized curves can be found in the Figure 6 legend. Although this correspondence is certainly less rigorous than in the human setting, this is to be expected considering the modeling process of Hay et al. (2011) was distinct from that of Rich et al. (2021), including a reduced emphasis on capturing h-current-driven subthreshold activity; this is also a likely explanation for the idiosyncrasy that the rodent model FDG curves are identical with and without h-current activity. Beyond a different emphasis on h-current-driven dynamics, the model of Hay et al. (2011) is constrained to exhibit calcium spikes and backpropagating action potentials, which the human model is not; this might explain differences in the suprathreshold spiking frequency preference measured by the FDG. Nonetheless, there is sufficient correspondence between the *in silico* and *in vitro* settings to reasonably justify using this model in similar analyses as performed on the human model to assess the relationship between ion channel contributions and FDG properties in the *in silico* setting.

We note that the minimal effects of h-channel blockade on the spiking frequency preference of rodent L5 neurons, either using ZD-7288 *in vitro* or by making the Ih maximum conductance zero *in silico*, echoes the existing literature showcasing a reduced effect of ZD-7288 in some rodent neurons. In a comparative study of rodent and human L2/3 cortical pyramidal neurons, Kalmbach et al. (2018) found application of ZD-7288 had a notably diminished effect on rodent neurons relative to human neurons, including no appreciable change in the resting membrane potential (RMP) of rodent neurons and a diminished change in their input resistance. The work of Kalmbach et al. (2018) highlights a potential explanation for these differential effects across species via the increased expression of HCN1 in human neurons compared with their mouse counterparts across cortical layers. We found that although application of ZD-7288 caused a significant (*p <* 0.05, two-sample *t* test) decrease in the sag voltage and significant increase in the input resistance in our rodent L5 neurons, conforming with previous results (Hu et al., 2002; Dembrow et al., 2010; Schmidt et al., 2017), it did not cause a significant change in other intrinsic properties including the RMP and rheobase. Meanwhile, in our human L5 neurons, there is a statistically significant hyperpolarization of the RMP following application of ZD-7288 (−67.53 mV to −70.83 mV; *p <* 0.05, two-sample *t* test) and decrease in the sag voltage; this parallels what was observed in our model neuron, whose RMP decreases from −72.4 mV to −83.7 mV with h-channel blockade. Together, these results illustrate that the funny (DiFrancesco and Borer, 2007) nature of the h-current combined with potential secondary effects of ZD-7288 (Poolos, 2004; Sánchez-Alonso et al., 2008; Chevaleyre and Castillo, 2002) make deriving its specific role in complex neuronal dynamics especially difficult to assess, providing additional motivation for the interdisciplinary approach adopted in this study. These results also provide additional evidence that the effects of ZD-7288 may play a subtly different role in shaping neuronal activity across species.

With the appropriateness of the Hay et al. (2011) model thus justified, we applied our STA/Currentscape analysis to analyze the contribution of inward currents, particularly the h-current, to neuronal dynamics in the moments before spiking. This reveals notable differences in the Currentscape STAs of the rodent model compared with the human. First, the primary contributors to neuronal dynamics are the Na_Ta_t and Ca_LVAst currents (as opposed to the Na_Ta_t and h-currents in the human model), both of which have largely monotonic dynamics on qualitative inspection in the 200 ms before spiking. Second, the contribution of the h-current is notably smaller, meriting the magnification presented in Figure 6*F*. Finally, the contributions of the Nap_Et2 and Ca_HVA currents are smaller by approximately another order of magnitude and largely negligible.

The dynamics of the contribution of the h-current appear to be more mononotonic than in the human model on qualitative inspection. We confirmed this using the same quantification as performed on the human model, finding that $I_{*}^{Ih}$ decreases for 85.85% of the STA time series, notably more often than the 62.50% in the human setting.

Implicit in the overall hypothesis of this work, that the distinguishing features of the contribution of the h-current to neuronal activity are necessary for the distinct features of the FDG of human L5 pyramidal neurons, is the inverse; neurons with different h-current dynamics should not exhibit these FDG properties. Our analysis of the Hay et al. (2011) model and rodent *in vitro* results showcases exactly this phenomenon; rodent L5 pyramidal neurons lack low-frequency FDG peaks both *in vitro* and *in silico*, which corresponds with a diminished and more monotonic contribution of the h-current to neuronal dynamics in the 200 ms before spiking. In essence, we here have adapted the strategy of proof by contradiction to support our main hypothesis via a convincing example in which the inverse of our argument holds.

## Discussion

In this study, we injected a noisy input current into a biophysically detailed, multicompartment model of a human L5 cortical pyramidal neuron, extracting precise characterizations of ionic current contributions to spiking activity that are not attainable with analogous *in vitro* experiments. This yields three major conclusions regarding the relationship between the h-current and the frequency preference of these neurons: (1) Disruption of h-current activity alone dissipates the low-frequency peak in the FDG that hallmarks the spiking frequency preference of these neurons; (2) the h-current exhibits distinctive activity in the 200 ms leading up to spiking, a previously undescribed phenomenon providing a viable explanation for the frequency preference of these neurons at this time scale and its dependence on h-current activity; and (3) differences in this frequency preference between human and rodent L5 pyramidal neurons correspond not only with distinct h-current kinetics between species (Rich et al., 2021; Kole et al., 2006), but also the activity of the h-current in physiologically relevant settings. This supports the previous hypothesis (Moradi Chameh et al., 2021), driven by *in vitro* experiments, that the contribution of the h-channel is necessary for the frequency preference of human L5 cortical pyramidal neurons and their leading role in human cortical theta oscillations (Florez et al., 2015; McGinn and Valiante, 2014).

The *in silico* setting is not only useful, but in fact necessary, to derive these conclusions, as it is not possible experimentally to simultaneously record the membrane potential of a neuron and multiple ionic currents in response to a complex input. This is particularly relevant for studying the h-current, as during *in vitro* current-clamp experiments, properties of this current are most commonly inferred from the sag voltage (Moradi Chameh et al., 2021; Kalmbach et al., 2018; Zemankovics et al., 2010; Guet-McCreight et al., 2021) rather than directly quantified. We fully exploited the opportunities presented by the *in silico* setting by combining a contemporary visualization tool, Currentscape (Alonso and Marder, 2019), with ubiquitous spike-triggered average analysis (Schwartz et al., 2006) to identify the average contribution of each ionic current, including the h-current, in the moments before spiking in response to a noisy stimulus. Our computational models allowed us to manipulate the ionic current contributions in a precise fashion, unlike what would be possible in *in vitro* settings with pharmacological blockade (which also typically has secondary effects). This facilitated our connection between the enhanced nonmonotonicity of the dynamics of the h-current when the Ih maximum conductance was doubled and the mitigation of this dynamic when this conductance was halved with corresponding changes in the FDG to further support our conclusions. As these were the only changes implemented in the model neuron, it is apparent that these changes to the h-current directly drive the observed changes in neuronal activity. Similar Currentscape/STA approaches have been used with a computational model of a hippocampal interneuron to show the underlying biophysical currents contributing to theta frequency spiking resonance (Sun et al., 2022).

The correspondence between the *in vitro* FDG (Moradi Chameh et al., 2021) and that of the model neuron of Rich et al. (2021) is vital and nontrivial. Given inherent limitations in the study of human cortical tissue, the model of Rich et al. (2021) was generated primarily by matching subthreshold voltage activity from a single neuron in a range with pronounced h-current activity, including adjusting the kinetics of the h-current. Spiking dynamics were constrained only by requiring the neuron to fire repetitively in an approximate frequency range exhibited by separate human L5 neurons in response to tonic inputs. The close approximation of the model of complex spiking dynamics observed *in vitro*, but not constraining model development, is additional support that the h-current plays a pivotal role in this activity.

We note that our analysis of the *in silico* model is limited to the soma to correspond to the *in vitro* experiments. The frequency preference of dendrites is a rich topic for future research, given both the nonuniform distribution of the h-channel (Rich et al., 2021; Hay et al., 2011) and unique morphologic properties of human neurons (Beaulieu-Laroche et al., 2018). We also acknowledge the limitations inherent in all computational modeling studies; the model neurons studied here are necessarily abstractions of the biophysical reality that cannot capture the entirety of the intricate dynamics of a neuron (Almog and Korngreen, 2014). Nonetheless, the focused conclusions of this study on the relationship between the h-current and spiking frequency preference are well justified given the specific correspondences between the *in vitro* and *in silico* settings in h-current activity and FDG. Given neuronal degeneracy (Edelman and Gally, 2001), it is possible that combinations of other ionic currents might affect the FDG properties related to h-current activity in this study; however, that does not affect the primary conclusion of this work, that that the h-current is a necessary contributor to these FDG properties for this well-developed model of a human cortical L5 neuron. Developing and exploring additional models and model populations is outside the focused scope of this article.

We have shown here that h-channels are essential in determining the spiking frequency preference of human L5 pyramidal neurons, which in turn implies that they can be key controllers of oscillatory activity produced by neuronal circuits. As more human data have become available, computational models of human cortical circuits have been developed and used to show how reduced inhibition in depression affects stimulus processing (Yao et al., 2022), how reduced neuronal heterogeneity can impair seizure resilience (Rich et al., 2022), and that age-dependent sag current increases affect resting state activities (Guet-McCreight et al., 2021). Although circuit models are needed to link to system level experimental data, there are many more experimental unknowns that need to be considered in developing such models. Indeed, exploration goals using model neuronal circuits often differ from those of studies using detailed cellular models of individual neurons. This work is an example of the latter, focusing on whether and how the specifics of particular channel types might affect complex cellular output (i.e., spiking frequency preference) that influence circuit output (i.e., rhythms). The detail needed in any model naturally varies with the question, and there is no clear consensus of the extent of experimental detail needed as we consider the multiscale nature of the brain (D’Angelo and Jirsa, 2022). Given these challenges, clarity regarding model generation and the goals of computational studies are required to accelerate our understanding of the brain (Eriksson et al., 2022) and usher interdisciplinary neuroscientists toward jointly tackling the challenges of neurodegenerative disease at cell and circuit levels (Farrell et al., 2019; Gallo et al., 2020; Rich et al., 2022).

## Synthesis

Reviewing Editor: William Stacey, University of Michigan Decisions are customarily a result of the Reviewing Editor and the peer reviewers coming together and discussing their recommendations until a consensus is reached. When revisions are invited, a fact-based synthesis statement explaining their decision and outlining what is needed to prepare a revision will be listed below. The following reviewer(s) agreed to reveal their identity: Nikolai Dembrow. Note: If this manuscript was transferred from JNeurosci and a decision was made to accept the manuscript without peer review, a brief statement to this effect will instead be what is listed below.

The revised paper is acceptable, but please just add units to the following sentence:

“standard deviation of 0.04 and τ = 3.”
